# Assessing Stigma Toward Mental, Neurological, and Substance Use Disorders in Liberia: A Population Representative Study

**DOI:** 10.4269/ajtmh.24-0203

**Published:** 2024-09-03

**Authors:** S. Benedict Dossen, J. Mike Mulbah, April Hargreaves, Samhita Kumar, David Mothersill, Gerard Loughnane, Eve Byrd, Angie Tarr Nyakoon, Joseph S. Quoi, Ikenna D. Ebuenyi

**Affiliations:** ^1^ The Carter Center, Monrovia, Liberia;; ^2^ Psychology Department, School of Business, National College of Ireland, Dublin, Ireland;; ^3^ The Carter Center, Atlanta, Georgia;; ^4^ Ministry of Health, Monrovia, Montserrado County, Republic of Liberia;; ^5^ Department of Rehabilitation Science & Technology, University of Pittsburgh, Pittsburgh, Pennsylvania;; ^6^ IRIS Center, School of Nursing, Midwifery & Health Systems, University College Dublin, Dublin, Ireland

## Abstract

Mental health stigma remains a major global problem associated with low self-esteem, social withdrawal, and poor health-seeking behavior in individuals. However, limited published evidence details these challenges in Liberia. Knowledge of public perceptions toward mental illness and key trends in the associations between knowledge of mental, neurological, and substance use disorders (MNSs) and stigma is crucial to designing evidence-based mental health policies and supporting service delivery. This population-representative survey explored and quantified stigma related to MNSs in four health regions in Liberia, using a multistage stratified random sampling of 1,148 residents. Four internationally validated scales were used to assess knowledge, attitudes, and perceptions toward schizophrenia, bipolar disorder, epilepsy, and substance use disorder including the 1) Mental Health Attribution Questionnaire; 2) Five Question Stigma Indicator Questionnaire to assess Community Stigma; 3) Reported Intended Behavioral Scale; and 4) Personal Acceptance Level of Conditions. Data from interviews with 1,140 participants (96% response rate) were analyzed using central tendencies, hypothesis testing with simple logistic regression, and bivariate analysis for association between dependent and independent variables. Low mental health knowledge was found to be a strong predictor of discriminatory behaviors and stigma. Results revealed that exposure to movies or television significantly predicted increased discriminatory tendencies and that a lesser degree of acceptance was shown toward substance use disorder than any of the other conditions. These findings underscore the need for increased awareness and education about mental health to eliminate stigma and promote better care and inclusion for people living with MNSs.

## INTRODUCTION

Conceptualizations of stigma as it relates to mental health and healthcare are multifold. The WHO describes social stigma as the negative association between individuals or groups with specific characteristics and a particular mental illness.^1^ A systematic review and meta-analysis examining the association between mental health–related stigma and active help-seeking identified four types of stigma: perceived public stigma, personal stigma, self-stigma, and attitudes toward help-seeking, finding a negative association between stigma and active help-seeking, with self-stigma having the most substantial effect.^2^ Although perceived public stigma, personal stigma, and self-stigma may appear to be clearer on face value, attitudes toward help-seeking as a type of stigma may be confusing, in that it could also be seen as an outcome of stigma. For clarity, attitudes toward help-seeking are basically help-seeking stigma or the emotional reaction or position toward people who seek help for mental health problems. This stems from a notion that people who seek help for mental health problems are weak. So, although stigma against people with mental health problems may hinder them from seeking help, the attitudes of other people toward them can also prevent them from seeking help. Also, Gonzales et al.^3^ referred to community stigmas as the stigmatizing beliefs and attitudes held by members of a community toward mental illness, whereas Thornicroft et al.^4^ framed stigma as something that encompasses three key elements: problems of knowledge (ignorance), problems of attitude (prejudice), and problems of behavior (discrimination). Taking these frameworks together, three types of stigma tend to play a significant role in mental health: 1) public stigma, which involves negative or discriminatory attitudes that others have about mental illness; 2) self-stigma, which refers to the negative attitudes, including internalized shame, that people with mental illness have about their condition; and 3) institutional stigma, which involves systemic practices or policies of government and private organizations that intentionally or unintentionally limit opportunities for people with mental illness.

### Mental health and mental disorders.

Mental health and mental disorders exist on a spectrum and are a function of biological, social, and environmental factors. Defined as “a state of mental well-being that enables people to cope with the stresses of life, to realize their abilities, to learn well and work well, and to contribute to their communities,” mental health is an integral component of health and well-being and more than solely the absence of illness.^5^ The WHO identifies many types of mental disorders, defined in accordance with the *International Classification of Diseases 11th Revision*. As of 2019, a staggering 970 million people, or approximately one in every eight individuals, were reported to be living with such conditions globally, among which anxiety and depressive disorders emerged as the most prevalent and highest causes of disability and dependency.^5^

According to a 2015 report by the WHO, approximately 10% of people in a given population suffer from common mental health disorders such as mild-moderate depression, anxiety, and alcohol and substance misuse.^5^ In addition, 3% of the population experiences severe mental illnesses such as schizophrenia and bipolar disorder.^6^ In Liberia, which has a population of more than four million, over 400,000 individuals were estimated to have some form of mental disorder, with roughly 130,000 experiencing severe mental illnesses such as schizophrenia, bipolar disorder, and post-traumatic stress disorder.^7^ Subsequent estimates showed that one in five Liberians suffer a mild to moderate mental disorder.^8^ Building on a global prevalence rate for mental illness of 13%, modeling efforts suggest that by the end of 2022, about 604,688 people in Liberia were suffering from one or multiple mental, neurological, and substance use disorders (MNSs).^5^ These statistics were likely understated owing to the country’s 14-year-long civil war, the 2013–2016 Ebola virus disease outbreak, and the COVID-19 pandemic. In the wake of social stigma, lack of necessary services, maltreatment, and harmful social beliefs, individuals with MNSs often face abandonment or even loss of life.^9^

#### Epilepsy.

Brain health, much like mental health, exists on a continuum that ranges from well-being to disorders and disability. The consequences of poor brain health are far-reaching and can result in negative outcomes, including morbidity and mortality. Among neurological or brain conditions, epilepsy features prominently in the disease burden of low- and middle-income countries. Epilepsy is a chronic disease characterized by recurrent seizures, sometimes accompanied by loss of consciousness and control of bowel or bladder function. It affects around 50 million people worldwide.^10^ In Liberia, epilepsy accounts for 50% or more of MNS cases reported by health facilities that provided service delivery data to the Liberian Ministry of Health in the last year.^11^

### Substance use disorders.

According to the American Psychiatric Association, substance use disorder (SUD) is “a complex condition in which there is uncontrolled use of a substance despite harmful consequences.”^12^ People with an SUD have an intense focus on using certain substances, such as alcohol, tobacco, or illicit drugs, to the point where the person’s ability to function in day-to-day life becomes impaired. As many as 11% of adults have experienced an SUD in Liberia.^13^ Many of these disorders are believed to have stemmed from various impacts of the Liberia Civil War, the COVID-19 pandemic, and ongoing poverty among the population.^14^ Over time, SUDs can contribute to social isolation and shame. These challenges can directly impact emotional, physical, and mental health. Substance users are often separated or disowned by family members. Embarrassment, shame, and even financial challenges regarding their condition can limit their access to healthcare resources.^15^

### Study significance.

Stigma can have detrimental effects on individuals living with MNS conditions, as well as their families, caregivers, friends, and communities.^1^ More than half of people with mental illness do not receive help for their disorders because of stigma, prejudice, and discrimination against people with mental illness as a result of a lack of understanding or fear.^16^ Inaccurate or misleading media representations of mental illness exacerbate these challenges.^17^^,^^18^

There is a small but growing evidence base indicating that reported stigma among individuals with MNS disorders in low-income countries is greater than that in high-income countries.^19^ Liberia has not conducted a comprehensive national prevalence study, and there is a dearth of information regarding the prevalence of stigma and discrimination related to mental illness, epilepsy, and substance use in the country. Therefore, there is a need for research on mental health–related stigma as it pertains to the Liberian context. Researchers, policymakers, and stakeholders must prioritize the collection of localized evidence to address the gaps in public knowledge and perception surrounding mental health.

This study therefore assesses these critical aspects of stigma (e.g., the types of stigma, the knowledge/information gap, and its impact) to better appreciate its form and magnitude in Liberia. As part of a larger cross-country analysis assessing knowledge, perception, and attitudes toward mental, neurological, and substance use disorders in Liberia and Ireland, the issues considered in this study are schizophrenia, bipolar disorder, epilepsy, and SUD with emphasis on alcohol, tobacco, and illicit drug use.^20^ Owing to the shared volume of cases reported and the national health security implications, these four conditions are emerging as MNS issues of public health concern and are therefore preferred. In the context of Liberia, this study aimed to evaluate 1) how knowledge of MNS conditions influences behavior and attitudes toward people with MNS conditions and 2) the social predictors (e.g., level of income, level of education, geographical location, standard of living) of behavioral responses to individuals living with MNS conditions, with the hypothesis that increased knowledge of MNS conditions correlates with lower levels of expressed stigma toward persons with mental illness.

## MATERIALS AND METHODS

This cross-sectional study was carried out between May and October 2022 by The Carter Center Mental Health Program in Liberia, the National College of Ireland, and the University College Dublin. This study follows similar themes and analyses from a study conducted in Ireland, which was based on a study conducted in France by Durand-Zaleski et al. and others.^20^^,^^21^ This study was partially sponsored by Esther Ireland, an Irish Aid health development cooperation initiative and member of the ESTHER Alliance for Global Health Partnerships.

Persons targeted included individuals aged 18 years and older living in Liberia, constituting 51.8% (1,799,840) of the population.^22^ A total of 1,170 persons were targeted to be interviewed across 63 clusters in four of the five health regions: North Western region: Bomi, Grand Cape Mount, and Gbarpolu counties; South Central: Montserrado, Margibi, and Grand Bassa counties; North Central: Bong, Nimba, and Lofa counties; and South Eastern A: River Cess, Sinoe and Grand Gedeh counties. South Eastern B region, comprising River Gee, Grand Kru, and Maryland counties, was excluded from the sampling frame because of inaccessibility, as the survey was conducted during the peak of the rainy season.

### Sampling.

The study used a three-stage, stratified, random sampling design in which, in the first stage, 63 clusters were randomly drawn from 13,596 localities across four of the five health regions based on probability sampling proportionate to the population size of each health region. The combined population of the 63 clusters represented 14% of the total population in the sampling universe. In the second stage, households were systematically sampled from within each cluster, the sampling interval being determined by the size or number of households in each cluster. Nine households were systematically sampled based on a random start in each cluster and interviewed to yield at least 18 valid respondents per cluster. Because most households in Liberia were not systemically categorized, random selection was done in the field jointly by a team of research assistants (RAs) and mental health clinicians (supervisors) to select the first households via a random start method (spinning of a pencil). The average household size is 5.1, and there are about 670,294 households within the general population.^22^ In the third stage, at most, two residents of each sampled household were interviewed.

The sampling interval was community specific based on the number of households and town size. At least one inhabitant aged 18 years or older was interviewed in each household. The next nearest household was automatically selected if a sampled household did not meet the age eligibility criteria. The first household was randomly selected in each cluster or community based on random start, and subsequent households were selected using a systematic sampling method. Each potential respondent was interviewed separately, and when there were more than two adults or eligible respondents, a second person was randomly selected and interviewed. Households with multiple adults (aged 18 or older) were also included in the third stage. From a given household, a list of potential respondents was drawn, resulting in a random selection. The interviewers used a lottery system by writing the initials of all eligible respondents on separate pieces of paper and then asking someone else to select one piece of paper without seeing the names. To minimize bias in selecting respondents, RAs were oriented to the overall study protocol, and supervisors monitored to ensure adherence.

The sample size was calculated using the following formula: *n* = [*EZ* 2 *p*(1-*p*)]/d^2^, where

- *E* = design effect accounting for a cluster survey design (i.e., 1.2);
- *Z* = 1.96 (for 95% CI);
- *p* = expected proportion with the characteristic of interest (31% adjusted downward from 31.2%^[^1^]^); and
- *d* = half the desired width of the CI.

Based on the context of Liberia, we anticipated a general MNS prevalence where 31% of individuals interviewed would have the characteristics of interest. Our desired precision for this estimation was set at 10%. It is important to note that the design effect, which represents the variation in sampling error, can differ significantly from survey to survey and even within the same survey. For this calculation, we assumed a design effect of 1.2, indicating a 20% variation in sampling error compared with the expected under simple random sampling. Using these values, we determined that a sample size of 570 households was required, and assuming an average of two potential interviews per household, we estimated that a total of 1,180 interview sessions or individuals would be interviewed. This robust sampling approach taking into consideration the sample size, regional consideration, clustering, and randomized selection of study participants would ensure the representativeness of our sample relative to the national population.

### Ethics review.

Official approval for the study and tools was obtained through consultations with national and local authorities. At the national level, three formal in-person presentations were done by the Principal Investigator. The first presentation was at the level of the Liberian Ministry of Health for official approval and endorsement of the study. This presentation was made to the Minister of Health and the Director of the Mental Health Unit. The second presentation was done for the Technical Coordinating Committee on Mental Health, the official body that advises the Minister of Health on mental health, neurological, and substance use issues in Liberia. After both presentations, the Minister of Health officially endorsed the study, paving the way for the third presentation to the ethics board (Atlantic Center for Research and Evaluation Institutional Review Board [ACRE IRB]), Protocol #22-02-304/Assurance #FWA00004853. At the community level, apart from the official approvals from the Liberian Ministry of Health and the ACRE IRB, the study team worked closely with the County Health Teams of the selected counties in the four regions. The County Health Teams have strong community-based structures. Through these structures combined with the expertise and experience of the mental health clinicians, we connected with local leaders and community members to obtain local acceptance to conduct interviews in the communities. In practical terms during data collection, the field supervisors (clinicians) liaised with the appropriate health supervisors assigned in the districts or counties to lead the charge of introducing the team and the purpose of their mission to the community leadership and obtained their permission to conduct the survey in their respective catchment areas.

### Fieldwork.

A two-day training was conducted for four RAs and field supervisors on the study methodology, application and administration of tools, research principles, and human subject protection. The training culminated in field testing of methods and tools and subsequent debriefing sessions. Four locally licensed mental health clinicians were invited to assist the RAs in defining key mental health terminologies (based on the local context) and delivering the tools professionally and sensitively. Each team was assigned specific enumeration areas or clusters in specific regions. Montserrado, being the most proximate and most populated county with the highest number of clusters, was engaged during the early days of fieldwork by all teams, each covering a designated number of clusters. This approach provided further opportunity for rigorous supervision of the data collection process and to ensure the quality of adherence to survey methodologies and protocol when more remote regions were sampled.

All the scales used for the study were simplified in simple English to ensure they were contextually and culturally relevant and feasible. This means that idioms, big terminologies, or languages that are not common in day-to-day transactions by the average Liberian, especially those in rural areas, were replaced with simpler equivalents (words, phrases, or clauses). Hands-on exercises and field testing characterized the translation, and feedback sessions were convened for further adaptation if necessary. We reviewed all the scales for language clarity and accuracy as well as interviewees’ receptivity, considering the sensitivity of the questions about mental health, epilepsy, and SUDs. The clinicians helped in explaining the mental health disorders (e.g., schizophrenia and bipolar) using appropriate and familiar local idioms to enhance the comprehensibility and acceptance of the RAs. After the revision, the scales were digitized and published to a mobile application to facilitate quality assurance and real-time monitoring of the data collection. During data collection, clinical terminologies such as epilepsy, bipolar disorders, schizophrenia, and SUDs were further translated or communicated to interviewees in the local parlance (Liberian English) by the mental health clinicians, who were experienced through their clinical practice and adept with depicting associated symptoms, manifestations, etc., of the conditions. Liberian English is a form of pidgin widely spoken around the country.

### Measures.

The study used globally validated mental health assessment tools to assess community stigma, mental health knowledge, perception, attitude, and behavioral practices toward individuals with severe mental health conditions. Mental health knowledge was the independent variable, and MNS-related stigma was the dependent variable. Considering that stigma has been shown in other studies to be driven by a lack of understanding or misinformation, exploring the level of mental health knowledge and perception of MNS that individuals possess can provide insight into the underlying factors that contribute to stigmatization of MNS. This can allow researchers, policymakers, and other actors to develop effective interventions to address gaps in knowledge and understanding, thereby reducing stigma and improving MNS outcomes.

The tools, including the Mental Health Attribution Questionnaire (AQ9), the 5 Question Stigma Indicator – Community Stigma (5QSI-CS) tool, and the Reported and Intended Behavior Scale (RIBS) assessment tool, were digitized and deployed to portable handheld computing devices (Samsung Galaxy Tab A7 tablets) for real-time data collection.

The AQ9 is a nine-item self-reported assessment tool designed to measure public stigma toward people with mental illnesses. It assesses emotional reactions and discriminatory responses based on hypothetical vignettes. It rates public stigma on a 9-point Likert scale ranging from 1 = “Not at all” to 9 = “Very much” and scored each of the nine constructs by computing or summing the responses to the items comprising that construct, for a maximum score of 81. Higher scores represent greater endorsement of the corresponding attitude or belief.

The 5QSI-CS is used to assess levels of community stigma and is generally used in the field of neglected tropical diseases. The tool scores responses to five questions concerning key life areas of discrimination, concealment, shame, social distancing, and avoidance on a scale using never (0), sometimes (1), often/usually (2), and don’t know (0). The lowest score is 0, and the highest score is 10.

The RIBS has eight questions and is based on the Star Social Distance Scale to assess reported (present and past) and intended (future) behavioral discrimination against people with mental health issues. Items 1–4 of the RIBS assesses self-reported behavior based on four themes (living with, working with, living nearby, and having a close friend experiencing a mental health problem). The binary response options are coded with 1 for “yes” and 0 for “no.”

Our decision to include the RIBS was based on it being one of a few validated psychometrically tested instruments for testing behavioral discrimination and analyzing the presence of reported and intended stigmatizing or discriminatory behaviors against people with mental health problems in the general population. In addition, not only is it brief, feasible, and psychometrically robust for assessing mental health–related reported and intended behavioral discrimination, but it is also helpful for evaluating the effectiveness of interventions intended to reduce stigma and/or discrimination related to mental illness.^23^^–^^25^

We assessed basic knowledge about mental health within the general population by asking open-ended questions about general classification, causes, predispositions or risk factors, treatment advice, etc. Where necessary, respondents were prompted to respond to specific themes in our line of general questioning about mental health. In our analysis, we coded responses for the basic mental health knowledge questions into correct and incorrect knowledge based upon prevailing clinical and theoretical guidance on each thematic concern featured in the general knowledge questions. The general knowledge question in which respondents were requested to say all they know about mental health (open-ended) and the resulting response sets were coded based on the following themes: (definition, scope, causes, manifestation, risk factor, prevention, treatment [options and guidance], health-seeking or help-seeking advice, effects of MNS, etc.). Correct responses for each theme were coded as a 1 and incorrect responses as 0. For example, if a respondent said that “Many people have mental problems but do not realize it” or “Mental disorders are caused by incorrect thinking” or “Mental disorders and psychological problems cannot be prevented,” such responses were gauged against clinical guidance and coded accordingly. Similarly, close-ended questions were coded for the knowledge question about specific MNS conditions (epilepsy, schizophrenia, bipolar disorder, and SUDs), and the questions asked were related to the characteristics or manifestations of the conditions. For example, whether the condition is contagious, gets worse with time, can be diagnosed early in life, is a condition with which one can live normally with treatment, involves lifelong treatment, is hereditary (genetic), requires lifelong hospitalization, causes motor disabilities, or first appears in young adults (i.e., age of onset). The response sets are strongly agreed, agreed, neutral, disagreed, strongly disagreed, and don’t know. Correct responses were coded as 1 and incorrect responses as 0.

Subsequently, to assess the depth and scope of knowledge, we asked about the characteristics or manifestations of specific MNS conditions. The questions asked included the extent to which respondents agreed or disagreed with specific propositions about schizophrenia, bipolar disorder, epilepsy, and SUDs concerning whether the disorders are contagious, can get worse with time, can be diagnosed early in life, require long-term hospitalization, are hereditary, involve lifetime treatment, and more. The correct responses were then coded during our analysis and subsequently scored to yield a composite knowledge index score, which was essentially the summation of all nine dimensions of the question for each condition, the ideal score being 9, assuming a correct response for all nine domains assessed for each mental and neurological health condition.

Lastly, the study team assessed “Personal Acceptance Level of Condition,” based on the following three constructs or dimensions: 1) acceptance or willingness to live under the same roof with a person if they had a specific MNS problem; 2) willingness or readiness to allow one’s child to be in the same class as a person if they had a specific condition; and 3) willingness to work with someone with a diagnosis of the conditions. Each of the constructs earned a maximum of 2 points. The effect of the level of knowledge on behavioral and attitudinal outcomes was cross-referenced with findings from the RIBS, AQ9, and 5QSI-CS.

## STATISTICAL ANALYSES

In preparation for data analysis, data were exported from the web-based interface and input into Stata v. 16 (College Station, TX) for data cleaning and analysis. Basic descriptive analysis to describe the central tendency (mean, median, mode), dispersion (range, variance, and SD), and distribution of each dataset was conducted. Confidence intervals were derived at 95% confidence level by computing the net difference between the margins of error and sample mean for each observation. *P*-values were adjusted, considering the cluster sampling nature of the study design to correct for potential biases in parameter estimates and assess the internal consistencies of tests and measures, and we ran Cronbach’s α testing on each scale.

## RESULTS

The randomly interviewed 1,148 respondents met our required sample size of 1,140; they were from 725 households and represented a 96% response rate of the people we asked to interview.

### Demographic characteristics.

We explored a range of demographic questions about age, sex, locality, date of birth, education, income, household amenities, household size, role in household, etc. As shown in Table 1, the sample was comprised of 44.8% males and 55.2% females. The mean age of respondents was 36.7 years, with an average household size in terms of mean inhabitants per household as 5.9. Respondents were mainly self-employed (56.4%), unemployed (24%), and formally employed (15.8%), and only 3% were informally employed. Pertaining to household income, we asked about average monthly income and currency (in US and Liberian dollars) and about standard of living, and we asked about house material composition (made of concrete, roofed with zinc, availability of electricity or pipe-borne water, etc.).

**Table 1:** Characteristics of survey respondents

Demographic Variables	North Central Region	North Western Region	South Central Region	South Eastern Region
Mean Age (years)	41.3	41.3	42.2	42.4
Sex Ratio	0.8:1	1:0.6	0.8:1	0.82:1
Average Household Size	5.6	5.3	6.0	5.1
Minimum Post-Primary Education	68%	85%	71%	64%
Formally Used	24%	41%	20%	4%
Informally or Self-Used	76%	59%	80%	96%
Average Monthly Income in US$	$113.42	$168.33	$108.01	$102.94
Low Income (less than average monthly income)	68%	38%	57%	68%
Better Standard of Living*	46%	26%	46%	18%

* Defined by household access to at least three essential household utilities (radio, air conditioner, television, etc.)

### Awareness, knowledge, and perception of MNS conditions.

Awareness of MNSs was high (98.9%; 95% CI: 98.4–99.5%). Of the 1,148 respondents interviewed, 99% (1,136) demonstrated some level of awareness of MNSs; 47.7% (542) attributed the source of their awareness to either active engagement with persons with a known MNS condition (89.1% or 484); passive observation of cases of MNS, usually from a distance (10.7% or 58); or association with a case of MNS (63%). Twenty-two percent (120) of respondents attributed their awareness of MNSs to media exposure via television or movies.

**Figure 1.**
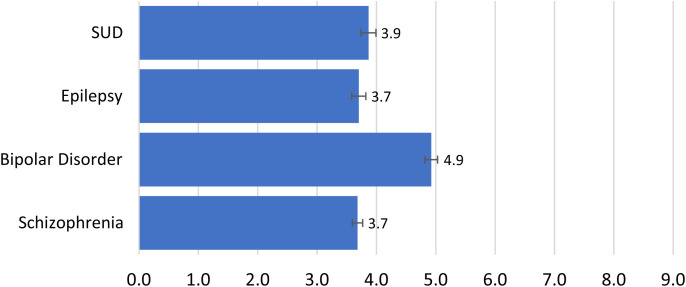
Average knowledge scores by MNS condition (Liberia, 2022). This figure depicts the sum of mean knowledge score for each MNS condition on the *y* axis, each computed as a composite measure of knowledge score derived from respondents’ knowledge and perception about the characteristics and manifestations of specific mental, neurological health, and substance use disorders. The *x* axis represents the average knowledge score, whereas the error bars in the chart represent the variation from the mean or point estimate at a 95% confidence level. MNS = mental, neurological, and substance use disorders; SUD = substance use disorder.

Conversely, MNS knowledge was low. Only 1.9% (CI: 1.1–2.7%) of the sample demonstrated strong knowledge (score ≥7), whereas 44% (CI: 14.5–47.3%) showed moderate to strong knowledge, with a score of (4 ≤*X* <7); 53.7% (CI: 50.8–56.5%) showed poor knowledge of MNSs (score ≤3). The MNS knowledge score was generally low for nearly all conditions. Although no single condition attained an ideal knowledge score of 9.0, the knowledge score for bipolar disorder was highest (4.9) compared with the other conditions (Figure 1).

Lower levels of MNS knowledge were significantly associated with the locality of residence. Urban residents were less likely than their rural counterparts to exhibit low knowledge of MNSs (odds ratio [OR] = 0.7; adjusted *P*-value <0.05). Similarly, age and sex were strong predictors of MNS knowledge endowment. Adolescents (aged 18–24 years) (OR = 0.081; adjusted *P*-value = 0.38) were less likely than their adult counterparts to exhibit low levels of MNS knowledge, whereas females (OR = 0.756, adjusted *P*-value = 0.012) were more likely than their male counterparts to exhibit low levels of MNS knowledge.

### Behavior toward persons with MNS conditions.

**Figure 2.**
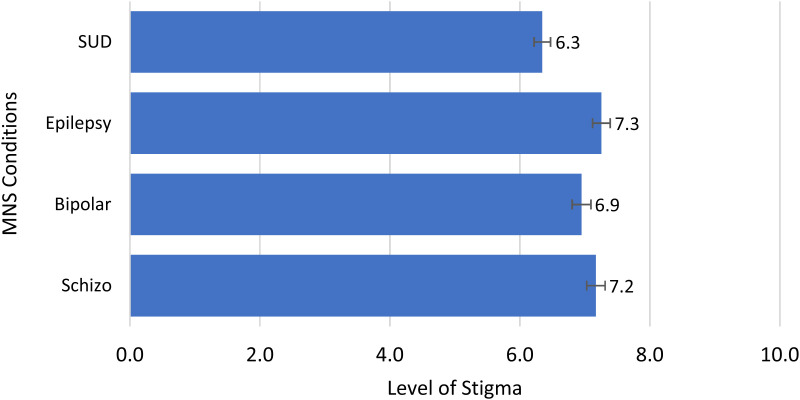
Levels of community stigma (5QSI-CS) by condition, Liberia (2022). Computed as the sum of values obtained from the administration of a five-question stigma indicator scale, each question on the scale represents a maximum of 2 points for each condition assessed. The conditions assessed are depicted on the *y* axis, whereas the sum of scores achieved for each condition is depicted on the *x* axis. The data show that there was a high level of community stigma associated with all the conditions assessed (*X* >6.2), but most notable were epilepsy (7.2) and schizophrenia (7.2). The error bars in the chart represent the variation from the mean or point estimate at a 95% confidence level. MNS = mental, neurological, and substance use disorders; Schizo = schizophrenia; SUD = substance use disorder.

In evaluating community stigma using the 5QSI-CS, results showed elevated levels of stigma (6.3–7.3) across MNS issues assessed (see Figure 2).

Epilepsy (7.3) and schizophrenia (7.2) scores were high for all five constructs, especially those associated with social distancing of persons with schizophrenia, bipolar disorders, SUDs, and epilepsy. Persons with SUDs were more likely than those with epilepsy, bipolar, and schizophrenia to be avoided, to have problems getting married, or to be stigmatized when it comes to finding a job, whereas those with epilepsy were more likely to experience internalized stigma than those with SUDs, schizophrenia, and bipolar disorders (OR = >3; adjusted *P* <0.05). They were also more likely than those with schizophrenia and bipolar disorders to be avoided (OR = >4; adjusted *P*-value <0.05) and nearly equally likely to have difficulties due to stigma in getting married or sustaining a marital relationship (OR = >3.5; adjusted *P*-value <0.05).

### Level of acceptance of people with MNS conditions.

**Figure 3.**
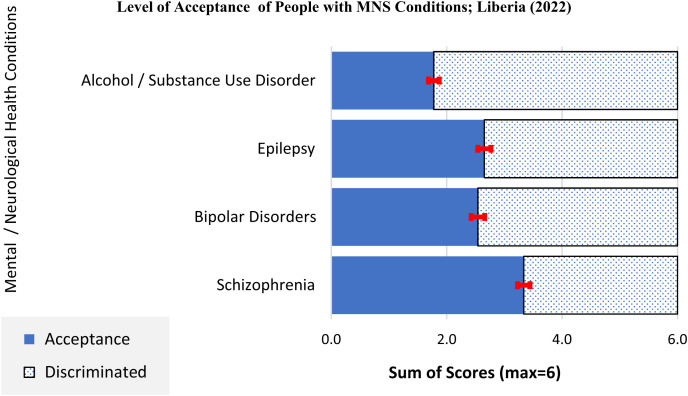
Personal acceptance level of condition (Liberia, 2022). The effect of public stigma on individuals with an MNS and how they could adversely impact personal perception and acceptance of the level of illness (internalized stigma) are depicted. Plotted on the *y* axis are the four MNS conditions assessed, and plotted on the *x* axis are the stigma index scores. The bars are distinguished in appearance; the dotted bar represents discrimination and the blue personal acceptance. Findings revealed a higher level of discrimination against persons with alcohol and SUDs and relatively higher sense of compassion toward persons with schizophrenia. MNS = mental, neurological, and substance use disorders.

Our findings revealed a high level of stigma against persons with all conditions but a higher level of stigma against persons with an SUD (4.2) and a relatively higher sense of compassion toward persons with schizophrenia (Figure 3).

**Figure 4.**
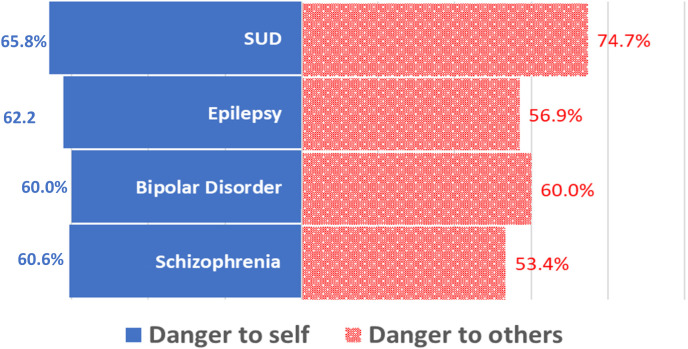
Evaluation of perceived danger (Liberia, 2022). The level of perceived danger associated with the four conditions assessed is depicted, with the red bars representing perceived danger to self and the blue bars representing perceived danger to others. With the associated percentage labels representing the magnitude of perceived danger, findings point to the perception of a relatively higher danger, both to self and to others, for persons with alcohol and SUDs than the rest of the conditions assessed. Noteworthy is the finding about the general perception of danger associated with all four conditions assessed. MNS = mental, neurological, and substance use disorders; SUD = substance use disorder.

Using the Levels of Acceptance of People with MNS Conditions scale to assess level of stigma, the three questions were asked for each of the four MNS conditions. A higher level of discrimination was found against individuals with an SUD (4.4) than with the rest of the three conditions assessed. We found a statistically significant association between level of MNS knowledge and levels of stigma toward persons with bipolar disorders and SUDs, such that a lower level of MNS knowledge was associated with higher levels of stigma against persons with bipolar disorders (OR = 1.5; adjusted *P* <0.05) and lower levels of stigma against persons with SUDs (OR = 0.45, adjusted *P* <−0.05). Further analysis showed that compared with “low-income earners,” “high-income earners” were less likely inclined to accommodate or accept persons with both an SUD (OR = 0.98; *P* = 0.647) and bipolar disorder (OR = 0.85; *P* = 0.02) and more likely to stigmatize persons with schizophrenia (OR = 1.16; *P* = 0.02) and epilepsy (OR = 1.1; *P* = 0.08). They believed that persons with an SUD represented a significant danger to others (OR = 1.1; *P* = 0.04) (Figure 4). The personal acceptance of level of condition, or PALoC, scale demonstrated acceptable internal consistency, with a Cronbach’s α scale reliability coefficient of 0.82.

### How knowledge or awareness of MNS influences behavior and attitude toward people with MNS conditions.

Our findings revealed that MNS knowledge and awareness influenced MNS behavioral response, the extent and magnitude of which varied with specific conditions.

Application of the RIBS to measure MNS stigma-related behaviors in the general public revealed that although not statistically significant, people who themselves have struggled with a mental health condition or have had the experience of being mentally ill (OR = 2.8) were more likely to be receptive to future or close affinity to persons with mental ill health, and the difference was statistically significant (*P* ≤0.05). Family members, relatives, or close neighbors and persons who have had a shared job experience with persons with mental illness (OR = 1.2) were more likely to be receptive toward accommodating persons with mental illness. Reliability of the scale was gauged at 0.823 using the Cronbach α measure.

Application of the Mental Health Attribution tool to measure public stigma toward people with mental illnesses revealed similar findings. The constructs of the AQ9 with the highest overall mean scores were pity (7.5), followed by help (6.4) and coercion (6.2); segregation and dangerousness were on par (5.3) with the overall average of all nine constructs combined (5), whereas anger (3.7), avoidance (4.3), and responsibility (4.3) rated below the mean score for all constructs combined. Reliability of the AQ9 scale was gauged at 0.663 using the Cronbach α measure (Table 2).

**Table 2:** Mental, neurological, and substance use attribution scores

Items	Dimension	Min	Max	Mean	p50	SD	Variance
	Mean AQ9 Score	2	9	5	5	1	2.0
I would feel pity for Harry.	Pity	1	9	7.5	9	2.2	4.9
I would be willing to talk to Harry about his problems.	Help	1	9	6.4	7	2.3	5.5
I would feel unsafe around Harry.	Dangerousness	1	9	5.6	6	2.9	8.3
If I were in charge of Harry’s treatment, I would require him to take his medication.	Coercion	1	9	6.2	6	2.3	5.4
Harry would terrify me.	Fear	1	9	5.3	5	2.9	8.6
I think Harry poses a risk to his neighbors unless he is hospitalized.	Segregation	1	9	5.6	5	2.4	5.6
I would think that it was Harry’s own fault that he is in the present condition.	Responsibility	1	9	4.3	4	2.7	7.3
If I were an employer, I would not interview Harry for a job.	Avoidance	1	9	4.3	4	2.6	6.6
I would feel aggravated by Harry.	Anger	1	9	3.7	3	2.5	6.4

Max = maximum; Min = minimum; p50 = 50th percentile.

Comparative analysis to assess the co-relationship between those who experienced or had knowledge of mental health stems solely from indirect contact with persons with an MNS condition on the one hand and those who, either by virtue of family or professional relationships, got to learn about MNSs from direct experience with persons with an MNS condition. The resulting behavioral outcome revealed a statistically significant association between lack of awareness about MNSs and public stigma against persons with an MNS. People who self-reported no awareness about MNSs were more likely to perceive persons with an MNS as being dangerous (OR = 2.1; adjusted *P*-value = 0.006). Persons attributing their source of awareness of MNS to the fact that they only got to know of MNS from rare experiences of observing a person with an MNS were more likely to perceive persons with an MNS as being dangerous (OR: 2.17; adjusted *P*-value = 0.009) or feared (OR = 4; adjusted *P*-value = 0.002). Close familiarity or relationship to persons with an MNS was associated with lower odds of public stigmatization (i.e., perceiving them as being dangerous) (OR = 0.4; adjusted *P*-value <0.005) or treated with fear (OR = 0.32; adjusted *P*-value <0.005).

Conversely, caregivers (OR = 0.27; adjusted *P*-value = 0.03) as well as family friends (OR = 0.63; adjusted *P*-value = 0.004) of persons with an MNS were less likely to treat them with fear or resentment. Limited or no prior familiarity with persons with an MNS was associated with higher odds of discriminatory behavioral response toward persons with an MNS illness. Against beliefs that persons with MNSs are usually to blame as being responsible for their illness, they therefore tended to endorse mental illness stereotypes and acted upon them in discriminating ways. People with reported experience or rare observation of persons with an MNS were more likely to hold them responsible for their condition (OR = 5.6; adjusted *P*-value = 0.004), whereas conversely, those who self-reported as having frequently observed (OR = 0.43; adjusted *P*-value = 0.004) cases of MNSs and family members of persons with an MNS condition (OR = 0.67; adjusted *P*-value = 0.050) were less likely to cast blame on persons with MNS conditions.

Limited personal interaction with persons with an MNS was also associated with higher odds of segregation (OR = 2.8; adjusted *P*-value = 0.034); avoidance of persons with an MNS condition (OR = 2.8; adjusted *P*-value = 0.033); fear of persons with an MNS (OR = 4.0; adjusted *P*-value = 0.002); perceiving persons with an MNS as being responsible for their condition (OR = 5.6; adjusted *P*-value = 0.004); and perceiving them as being dangerous (OR = 3.2; adjusted *P*-value = 0.009).

Our findings revealed that persons attributing their awareness of MNSs to media exposure, especially via watching movies, were twice as likely to perceive persons with an MNS as being dangerous (OR = 2.2; adjusted *P*-value = 0.039); thrice as likely to treat them with anger (OR = 3.7; adjusted *P*-value = 0.006), and albeit not statistically significant, more likely to adapt an avoidance posture toward persons with an MNS (OR = 2.5; adjusted *P*-value = 0.052). Conversely, although the likelihood of discriminatory tendencies remained high among persons exposed to MNS awareness through television shows, the differences were not statistically significant.

### Social predictors of behavioral responses to people with an MNS.

Our study found that locality (urban versus rural), standard of living, level of income, level of MNS knowledge, and level of education are strong predictors of behavioral response toward people with an MNS. The extent to which each social determinant influences behavioral responses varied in magnitude and direction with specific constructs as per the AQ9 scale. Urban residents (OR = 2; adjusted *P*-value = 0.023), residents of North Western (OR = 64; adjusted *P*-value <0.005), and residents of South Central (OR = 2.9; adjusted *P*-value <0.005) were more likely to perceive persons with MNS conditions as being dangerous, whereas those with a higher standard of living were found to be significantly less likely to stigmatize or discriminate across nearly all MNS behavioral constructs assessed. For example, people in the upper echelons of wealth were more likely to extend help (OR = 1.13; adjusted *P*-value = 0.006) or show compassion/pity (OR = 1.11; *P* = 0.051) for persons with mental illness. They were also more likely to support actions tantamount to coercion of affected persons to comply with treatment requirements. They were less likely to segregate or discriminate against persons with mental illness on the basis of fear (OR = 0.9; *P* = 0.23); avoidance (OR = 0.9; *P* = 0.04); anger (OR = 0.89; *P* = 0.04); segregation (OR = 0.96; 0.413); or the perception that they are responsible for their illness (OR = 0.95; *P* = 0.18) and dangerous (OR = 0.96; *P* = 0.201).

Our results further revealed that standard of living (OR = 7.48; *P* <0.005) and (Rural/Urban: OR = 0.16, *P* <0.005) and administrative regions (OR = 0.21; *P* = 0.009) were strong predictors of personal acceptance or reception toward persons with MNS conditions. Residents in the “Southeastern A” region were less likely to be receptive to persons with mental health conditions than their regional counterparts. Notwithstanding, though, people with mental health conditions were generally perceived as being dangerous both to themselves and to others; these perceptions were worse for individuals with an SUD in both circumstances (74.7% – danger to others; 65.8% – danger to self).

## DISCUSSION

Overall, this study suggests that stigma is a pervasive challenge in Liberia, posing a significant threat to the mental health and well-being of individuals with MNS conditions. This aligns with the 2016–2021 Mental Health Policy and Strategic Plan for Liberia, which acknowledges the widespread fear and misunderstanding surrounding individuals with mental illness as a prevalent issue.^26^ Our findings also align with other sources that MNS stigma is prevalent in Liberia and continues to hinder progress toward population mental health and well-being, despite recent improvements, such as the implementation of two mental health policies, the passage of mental health legislation, the establishment of a dedicated mental health budget, increases to the mental health workforce, and the integration of mental health into primary and community-based care.^2^^,^^27^^,^^28^ The 14-year civil war, Ebola outbreak, and COVID-19 pandemic have also profoundly exacerbated MNS challenges in the country.^29^ We found that the nature of the exposure (either to persons with mental illness or the quality of knowledge/awareness of mental health) matters largely, and our findings corroborate other research in this respect.

Our results showed that knowledge, experience, and socioeconomic status often intersect with perception, attitude, and behaviors regarding MNS conditions.^27^^,^^30^ Stigma encompasses problems of knowledge, attitudes, and behavior.^9^ We learned that those with family members with MSNs are less likely to exhibit discriminatory tendencies toward people with mental health conditions and perceive them as being dangerous or fear inducing, a finding largely consistent with those highlighted in the work of Corrigan and Nieweglowski,^31^ in which they postulated that “greater familiarity leads to less public stigma”.^32^ Also, people with better socioeconomic standing are less likely to exhibit discrimination based on fear, anger, and avoidance. This validates findings from a study by Wong et al.,^33^ in which low levels of stigma were identified among family members of persons with mental health disabilities. However, we also saw that people with better socioeconomic standing stigmatized those with an SUD and perceived them to be responsible for their condition. This relatively higher level of stigma and discrimination by people with better socioeconomic standing against persons with SUDs could be due to their personal fear about illegal activities or limited knowledge about the condition. People with better socioeconomic standing used the word “dangerous” when referring to those with SUDs. Their use of this word in relation to those with SUDs seemed to be figurative, alleging that involvement in illegal activities by persons with SUDs could negatively affect others who interact with them. Results also demonstrated how community stigma can significantly hinder the participation of those stigmatized within their communities, and the consequences can be far-reaching, impacting the overall well-being and quality of life of those affected. Community stigma can inhibit personal acceptance of conditions. The burden of community stigma can lead to shame, guilt, and isolation for people affected, creating barriers to accessing appropriate services and treatments and severe implications for their mental health outcomes. For instance, the results of the AQ9 indicated high scores for pity (7.5), help (6.4), and coercion (6.2). It was our impression that the high scores for these constructs may be indicative of concealed stigma toward people with MNS conditions (e.g., meaning that the pity exhibited may not be of compassion or empathy, but rather of disdain from community people or may be a reflection of the perception of alleged weakness or the perception of inferiority in those with MNS problems). Such “pity” or “attitude toward help-seeking” can influence the self-disclosure or help-seeking by persons with mental ill health.

Our findings revealed especially elevated levels of community stigma for epilepsy, schizophrenia, and SUDs. This was obvious across the five constructs of the 5QSI-CS: discrimination, concealment, shame, social distancing, and avoidance. Conversely, there was more compassion for people with schizophrenia than SUDs as indicated by the RIBS. This is intriguing and resonates with our anecdotal experience of neglect or avoidance of persons with an SUD against the perception of harm and danger. We found regional variations in the level of stigma across regions. From our results, Bong, Nimba, and Lofa combined (i.e., the North Central Region) were more likely to express stigma against persons with MNS conditions than the rest of the regions combined, and the difference between groups was statistically significant (OR = 1.8; *P* <0.05). This region of concern was arguably the epicenter of the civil war in Liberia and long-term tribal disagreements. Added to this is the general notion that mental illness is associated with witchcraft or is a justified penalty for retribution for heinous crimes committed against humanity during the war. Against this belief, there is usually less compassion for persons with mental illness, especially nonfamily members.

Our findings showed increased knowledge of SUDs. Specifically, respondents who reportedly observed persons with bipolar disorder, epilepsy, and SUDs were more likely to demonstrate a higher level of knowledge of bipolar disorder. Other studies suggest that settings that were exposed to major crises (e.g., war or disease outbreaks) tend to have high risk factors for incidence of bipolar disorder.^34^ Our study aligned with these findings, as we observed a statistically significant association between level and quality of knowledge of bipolar disorder and scope of exposure to persons with the condition. Liberia’s civil war and the devastating public health events (Ebola Virus Disease Outbreak, COVID-19 pandemic, and now the prevailing SUD crisis) have increased its people’s susceptibility not only to stress, trauma, and substance use problems, but also to bipolar disorder. Although our study did not purposely probe to understand the underlying factors for higher knowledge of bipolar disorder or SUDs compared with the other conditions, it is our professional assessment that the knowledge increase may be associated with the corresponding increase in conditions due to the civil war and public health events in Liberia, linked with the interaction of more people with the conditions at the community level. This implies that because people with bipolar disorder are not usually as extreme as those with schizophrenia, for which they would be generally hospitalized, there is a greater likelihood of people socializing with people with bipolar disorder in communities. Hence, people would be more familiar with the behavioral changes or outcomes of persons with the condition. Although people with SUDs are feared and avoided, they are more visible in the streets and there is a national appeal to respond to their crisis. On the other hand, people with schizophrenia are generally confined to mental health institutions, outcast to live as homeless persons in the streets, or blamed for witchcraft and demonic possession.

Our findings also revealed that mass media exposure (including exposure to movies and television) is associated with discriminatory tendencies and social distancing toward persons with MNS problems. Negative media portrayal of persons with MNSs often perpetuates misconceptions and stigmatization of mental illness. The unregulated projection of persons with MNSs as being violent, aggressive, dangerous, filthy, etc., in movies often can result in a discriminatory and stigmatizing attitude toward persons with MNSs. These findings, therefore, also have significant policy implications for media governance and regulation.

Our study has strengths and adds value to general stigma research, policies, and advocacy. We delved into the critical topic of stigma regarding public awareness and perception of individuals with MNSs in Liberia and examined the public’s attitudes and behaviors toward mental health situations. In addressing stigma and discrimination, we propose a comprehensive approach encompassing strategies targeted toward the various contributing factors, including being tackled at a systemic level to ensure that individuals receive the necessary support and resources to maintain good mental health and well-being. This includes training healthcare providers to deliver culturally sensitive and evidence-based care, a strategy that is aligned with the WHO’s Comprehensive Mental Health Action Plan (2013–2030)^35^ and Thirteenth General Program of Work (2019–2023),^36^ recognizing the critical role of the health workforce (including public health) in providing mental health services and emphasizing the need to invest in training and capacity building of the workforce to provide quality mental health services. In addition, this could involve collaborating with civil society organizations and other groups, such as journalists, people with lived experience from mental health, and school health clubs for community- and school-based mental health programs to advance advocacies, promote social inclusion, reduce stigma, support policy reform, or strengthen peer support and referral networks. We can advocate for comprehensive evidence-based strategies to reduce stigma and challenge the attitudes and beliefs that contribute to stigma by dispelling myths and misconceptions about MNSs and by promoting inclusivity and equality in healthcare settings. One such strategy could be The Carter Center’s behavioral health system strengthening program in Liberia covering workforce development, policy support, and social inclusion, which has been recognized for its potential in reducing stigma by the 2022 Lancet Commission on ending stigma and discrimination in mental health.^37^ Thus, by shedding light on public perceptions and quantifying stigma related to MNS conditions, immense contributions can be made toward policy reform, implementation of evidence-based interventions, and strategic advocacies, paving the way for better and more targeted interventions that address negative attitudes and behaviors toward individuals with MNS conditions in Liberia and more broadly in the West Africa region. Also, by addressing the underlying factors, such as improving access to education and employment opportunities, we can create an environment that is more supportive and understanding of individuals with MNS conditions.

This study has limitations with respect to design and bias. A cross-sectional study design limited the ability of the study to establish causal relationships between variables compared with a longitudinal study design. In addition, interviewer bias and social desirability may have influenced data collection, as interviewers’ own biases, assumptions, or interpretations of the interviews could have impacted the responses of the respondents. For example, we used Carter Center–trained mental health clinicians as supervisors to the RAs during data collection because of their vast experience working in the local context across most of the regions. As a bias-mitigating strategy to reduce potential influence of the supervisors (clinicians), we assigned them to different regions, other than their regular areas of work. Language bias of the instruments and associated with lower level of education may have also influenced participants’ responses. Similarly, to appear socially accepted, respondents may have answered differently to some questions that seemed to project a positive image of themselves. To mitigate the effect of these biases, we instituted several measures, including the involvement of interviewers in tool adaptation sessions, intensive training, and field testing of tools and methods, as well as rigorous supervision mechanisms during the entire data collection process.

Despite these limitations, the study did reach a 96% response rate, which was likely due to the approach to community entry, whereby we leveraged the influence of local health authorities (county health teams) and local health workers (district or county health supervisors and mental health clinicians) through joint inception briefings with community leadership in every major survey community to elicit the moral support of local community leadership for the maximum cooperation of study participants. Also, the quality of interactions between researchers and respondents and the clarity with which they communicated earned the confidence of the respondents.

## CONCLUSION

People with mental illness, epilepsy, and SUDs are often considered a potential danger to others or as being unable to function in society. These prejudiced beliefs against individuals with MNS conditions can lead to active discrimination that can impact them personally and economically in terms of their employment, education, housing, and healthcare. For the affected individual, this can contribute to low self-esteem and reduced treatment adherence; on a larger scale, this discrimination can extend to family, friends, and communities. Our study underscored the need for increased awareness and education about mental health to eliminate stigma and promote better care for people living with these conditions. Improving social relationships, addressing discrimination, and improving treatment access for individuals with mental health disorders are essential to improving their long-term health outcomes. There is a dearth of information regarding the prevalence of stigma and discrimination related to mental illness, epilepsy, and substance use in Liberia.

Taken together, our findings contribute to the evidence base by highlighting relevant information for policy, research, and practice regarding stigma against mental illness, epilepsy, and SUDs. Drawing from the findings, our study supports the importance of addressing education, poverty, social equity, and media stereotyping as critical factors in fighting stigma against MNS conditions. Considering the significant dearth of context-specific research on this topic, our study supports the need to generate more localized evidence to guide national policies. By doing so, we can enhance our understanding of the specific challenges and needs faced by individuals with MNS conditions and help them best by supporting systems tailored to their needs and local context. Ultimately, this can contribute to improving mental health outcomes and overall well-being in Liberia and globally.
